# Auto-titrating continuous positive airway pressure treatment for obstructive sleep apnoea after acute quadriplegia (COSAQ): study protocol for a randomized controlled trial

**DOI:** 10.1186/1745-6215-14-181

**Published:** 2013-06-19

**Authors:** David J Berlowitz, Najib Ayas, Maree Barnes, Douglas J Brown, Peter A Cistulli, Tim Geraghty, Alison Graham, Bonsan Bonne Lee, Meg Morris, Fergal O’Donoghue, Peter D Rochford, Jack Ross, Balraj Singhal, Jo Spong, Brooke Wadsworth, Robert J Pierce

**Affiliations:** 1 Institute for Breathing and Sleep, Austin Hospital, Melbourne, Australia; 2 Department of Respiratory and Sleep Medicine, Austin Hospital, Melbourne, Australia; 3 University of Melbourne, Melbourne, Australia; 4 Department of Medicine, University of British, Columbia, Canada; 5 Victorian Spinal Cord Service Austin Hospital, Melbourne, Australia; 6 University of Sydney, Sydney, Australia; 7 Department of Respiratory Medicine and Centre for Sleep Health & Research, Royal North Shore Hospital, Sydney, Australia; 8 Princess Alexandra Hospital, Queensland, Australia; 9 Stoke Mandeville Hospital, Mandeville Road, Aylesbury, Buckinghamshire, England; 10 Prince of Wales Spinal Medicine Department, Sydney, Australia; 11 LaTrobe University, Melbourne, Australia; 12 Department of Physiotherapy, Austin Hospital, Melbourne, Australia; 13 University of Otago and Burwood Spinal Unit, Christchurch, New Zealand

## Abstract

**Background:**

Quadriplegia is a severe, catastrophic injury that predominantly affects people early in life, resulting in lifelong physical disability. Obstructive sleep apnoea is a direct consequence of quadriplegia and is associated with neurocognitive deficits, sleepiness and reduced quality of life. The usual treatment for sleep apnoea is nasal continuous positive airway pressure (CPAP); however, this is poorly tolerated in quadriplegia. To encourage patients to use this therapy, we have to demonstrate that the benefits outweigh the inconvenience. We therefore propose a prospective, multinational randomized controlled trial of three months of CPAP for obstructive sleep apnoea after acute quadriplegia.

**Methods/design:**

Specialist spinal cord injury centres across Australia, New Zealand, the UK and Canada will recruit medically stable individuals who have sustained a (new) traumatic quadriplegia (complete or incomplete second cervical to first thoracic level lesions). Participants will be screened for obstructive sleep apnoea using full, portable sleep studies. Those with an apnoea hypopnoea index greater than 10 per hour will proceed to an initial three-night trial of CPAP. Those who can tolerate CPAP for at least 4 hours on at least one night of the initial trial will be randomized to either usual care or a 3-month period of auto-titrating CPAP. The primary hypothesis is that nocturnal CPAP will improve neuropsychological functioning more than usual care alone. The secondary hypothesis is that the magnitude of improvement of neuropsychological function will be predicted by the severity of baseline sleepiness measures, sleep fragmentation and sleep apnoea. Neuropsychological tests and full polysomnography will be performed at baseline and 3 months with interim measures of sleepiness and symptoms of autonomic dysfunction measured weekly. Spirometry will be performed monthly. Neuropsychological tests will be administered by blinded assessors. Recruitment commenced in July 2009.

**Discussion:**

The results of this trial will demonstrate the effect of nocturnal CPAP treatment of obstructive sleep apnoea in acute quadriplegia. If CPAP can improve neurocognitive function after injury, it is likely that rehabilitation and subsequent community participation will be substantially improved for this group of predominantly young and severely physically disabled people.

**Trial registration:**

Australian New Zealand Clinical Trial Registry ACTRN12605000799651

## Background

Spinal cord injury (SCI) is one of the most severe disabilities a person may sustain. The resultant loss of physical independence can lead to a significant requirement for assistance with personal care and activities of daily living, with consequent loss of privacy and compromised autonomy. The cost to the individual in terms of vocational opportunities and achievements can be extremely high. The cost to the community, in terms of lost work capacity, reduced ability to utilize prior education and training and the financial costs of disability pensions, carers’ pensions, attendant care, respite care, equipment and environmental adaptations, is associated with burden and reduced quality of life. In Australia, the annual incidence of SCI is approximately 15 per million [1,2]. Therefore, each year in Australia 260 people sustain a SCI and of these 57% will lose full function in their arms and legs (quadriplegia).

Quadriplegia affects 130 to 150 people per year in Australia [3]. The lifetime costs of the injury are substantial, even though the numbers affected are relatively small. In 2009, Access Economics estimated the lifetime healthcare cost of each incident case of quadriplegia in Australia to be $9.5million, with the total cost of SCI estimated at $2.0 billion [3]. The majority of SCI patients sustain their injuries in their second or third decade of life. If those who do not survive the first year following injury are excluded from analysis, then life expectancy approaches that of the general population [2]. Thus, if any secondary disease or impairment were a direct consequence of the SCI, it would have a significant effect for many years. Obstructive sleep apnoea (OSA) is such a condition.

The prevalence of sleep-disordered breathing, predominantly OSA, in quadriplegia is two to five times higher [4-11] than in the general population [12]; however, the reasons for this increased prevalence remain unclear. A prospective longitudinal examination of the sleep and breathing of all new patients with acute quadriplegia who attended a specialist spinal unit over an 18 month period found a prevalence of OSA of up to 83% in the first year after injury [13]. Sleep and respiratory studies were performed immediately after acute quadriplegia in 30 subjects (25 men) and at 2 weeks and 1, 3, 6 and 12 months post injury. Three subjects (10%) had probable OSA before their injury. However, 60% had OSA by 2 weeks after injury, 83% at 3 months and 62% at 1 year. These findings have been confirmed in another centre [14]. It is thus apparent that that the prevalence of OSA was extremely high in the first year after injury and that OSA is a direct consequence of acute quadriplegia.

Untreated OSA is a significant issue for those with quadriplegia. Previous authors have demonstrated that OSA in quadriplegia results in significant neurocognitive deficits [15]. Sajkov *et al*. demonstrated that hypoxia during sleep in subjects with quadriplegia and untreated OSA was associated with deficits in attention, concentration, memory and learning. Further, the neurocognitive impairments were both statistically and clinically significant in people with both quadriplegia and OSA, when compared with normal population values. These deficits are likely to prolong rehabilitation substantially, reducing future independence and limiting vocational outcomes following injury.

The usual treatment for OSA is continuous positive airway pressure (CPAP), which corrects sleep hypoxia and improves neurocognitive performance [16] and nocturnal blood pressure control [17] in the able-bodied with OSA. Reports of CPAP treatment in quadriplegia in uncontrolled studies are disappointing, with low levels of compliance with therapy reported. Stockhammer *et al*. [10] found 31 cases of sleep-disordered breathing after screening 50 subjects with quadriplegia. Only 16 of the 31 had previously used CPAP, with 11 (35%) continuing to use the device for at least a few weeks. Burns *et al*. undertook a cross-sectional postal survey of patients of a US Veterans Affairs Spinal Cord Service whose record indicated diagnosis or treatment for OSA [18]. Of those identified, only 39% were using CPAP at the time, while an additional 27% had used CPAP but had discontinued therapy owing to intolerance. In our previously described cohort study [13], five of the subjects were suspected clinically of having OSA and treatment with CPAP was offered. Only one of the subjects continued with CPAP for more than a few days, and that subject only did so after a period of respiratory failure. The reasons for this poor adherence are multifactorial, but the most commonly reported complaints are nasal congestion, an inability to fall asleep with the mask on and a lack of perceived benefit or noticeable change in symptoms.

In summary, it is known that OSA is a direct consequence of acute quadriplegia and that it is associated with cognitive deficits likely to impair rehabilitation after injury. Moreover, although CPAP is the usual treatment for OSA, it is poorly tolerated in quadriplegia. If the detection, treatment and adherence to CPAP therapy are to be improved in these patients, further research is vital. We propose to perform a prospective multicentre randomized controlled trial of CPAP for OSA after acute quadriplegia.

In preparation for this trial, the Melbourne research team completed a one-year feasibility study of this project [19]. The primary aim of the feasibility study was to determine the feasibility of CPAP treatment for OSA following acute quadriplegia. All patients (*n* = 44) with new quadriplegia who presented to the Austin Hospital during the nine months of study recruitment were eligible for study inclusion. Participants were tested for OSA with the Somté PSG (Compumedics, Abbotsford, Australia) and those with OSA were offered treatment with an auto-titrating CPAP device for three months. Rates of patient accrual, enrolment and OSA experienced during the study accurately reflected initial estimates. The feasibility study findings suggested that this current trial would confirm that OSA is associated with significant sub-acute morbidity and that CPAP treatment will be associated with an improvement in clinical outcomes.

The specific aim of the COSAQ study is to determine the effect of nocturnal auto-titrating CPAP treatment on neuropsychological function, quality of life, autonomic dysfunction and breathing in people with acute quadriplegia and OSA. To address this aim, one primary and two secondary hypotheses were developed and will be tested. The primary hypothesis is that usual care and nocturnal CPAP treatment will improve neuropsychological functioning more than will usual care alone; specifically, working memory as tested on the Paced Auditory Serial Addition Test (PASAT). The first secondary hypothesis is that the magnitude of improvement in neuropsychological function will be predicted by the severity of baseline sleepiness (Karolinska Sleepiness Scale (KSS)), sleep fragmentation (Sleep Efficiency, Arousal Index) and sleep apnoea (apnoea hypopnoea index (AHI), percentage total sleep time with SpO_2_ < 90%). The second secondary hypothesis is that usual care and nocturnal CPAP will improve the following parameters more than usual care alone:

1. Sleepiness and symptoms: Karolinska Sleepiness Scale (KSS) and the Basic Nordic Sleep Questionnaire (BNSQ).

2. Lung Function: spirometry.

3. Quality of life: Assessment of Quality of Life (AQoL).

4. Autonomic dysfunction: event diary and heart rate variability.

5. Health utility: AQoL-derived change in utility and associated quality-adjusted life years (QALY).

6. Depression and anxiety: Hospital Anxiety and Depression Scale (HADS) and the Profile of Mood States (POMS).

## Methods/design

### Funding

The trial is funded by the Transport Accident Commission of Victoria. The COSAQ study is an element of the ‘Sleep Health in Quadriplegia’ five-year programme grant.

### Design

A prospective multicentre randomized controlled trial will be undertaken. The control group will receive usual care and the experimental group will receive 3 months of nocturnal CPAP using an auto-titrating CPAP device. The trial is being conducted in 10 specialist SCI units in Australia, New Zealand, the UK and Canada. Ethical approval has been obtained from the Human Research Ethics Committee at each site and at the Austin Hospital (EC00204). Informed consent will be provided prior to recruitment and participation. Subject recruitment commenced at Austin Health in July 2009 and subsequently at the other sites, as they became ready. Recruitment is scheduled to finish mid-2015.

### Participants

Participants will be recruited from consecutive admissions at the trial sites.

### Inclusion criteria

•Acute, traumatic quadriplegia (T1 or higher lesion, complete or incomplete).

•Older than 18.

### Exclusion criteria

•Successful CPAP therapy for OSA prior to injury.

•Significant head injury (Glasgow Coma Score < 8 at first assessment).

•Ongoing hypercapnic ventilatory failure (PaCO_2_ > 45 mmHg at time of consent).

•Probable inability to be followed up until three months.

•Condition likely to significantly limit CPAP use (for example, major psychoses, facial or base of skull fractures).

### General testing procedures

**Figure 1:**
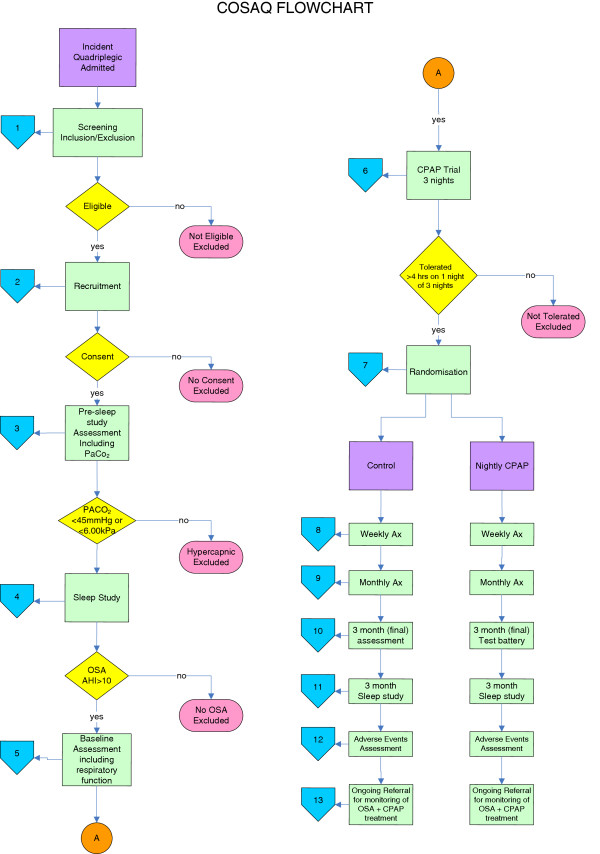
Flowchart of COSAQ study participant flow.

All testing will be performed at the participants’ bedsides. The respiratory function, questionnaires and other subjective data are collected at the same time in the mid-afternoon for each subject, to control for possible circadian influences. Sleep studies will commence at the subject’s usual bedtime. Figure 1 illustrates the study recruitment and data collection flowchart.

### Post-enrolment data collection

The listed information is collected at baseline following witnessed, informed consent. Human ethics approval has been provided for witnessed oral consent to be obtained where impaired upper limb function limits the ability of potential participants to provide written consent.

1. Demographic information (age at injury, sex, date of injury).

2. Time and date of assessment.

3. Current lesion level and completeness (Abbreviated Injury Scale score).

4. Medical history.

5. Height and weight.

6. Current medications.

7. Whether the subject has any intercurrent illness.

8. Likelihood of undiagnosed, pre-existing OSA (Multivariate Apnoea Prediction Index [20].

9. Abdominal girth at end-expiration and neck circumference.

10. Sleep studies.

The presence of OSA will be assessed using a portable sleep monitoring device, which comprehensively measures respiratory and sleep variables. (Compumedics™ Somté PSG, Abbottsford, Australia). All studies will be sleep-staged and respiration-scored by an independent trained sleep scientist. Sleep will be staged in 30-second epochs, arousals marked and respiratory events scored according to international standard criteria [21]. Summary indices and statistics will be calculated. Heart rate variability will be calculated from the ECG trace taken during quiet resting (prior to sleep onset).

All participants with an apnoea hypopnoea index (AHI) ≥ 10 will be considered as positive OSA cases and will proceed to have baseline measures (below) taken.

### Baseline measures

A number of tests are made prior to the trial of CPAP and randomization.

#### Cognitive test battery

•Rey Auditory Verbal Learning Test.

•Digit Span sub-test of the Wechsler Adult Intelligence Scale Revised.

•Paced Auditory Serial Addition Test (PASAT).

•Symbol Digit Modalities Test.

•National Adult Reading Test.

All these tests have been previously employed to show neurocognitive limitation in those with quadriplegia and OSA [15]. They are all administered orally, so that written replies or motor responses are not required. This battery of tests takes approximately 40 minutes to complete and measures function in the following areas: short-term memory, attention and concentration, immediate memory span, cognitive flexibility, internal scanning, working memory, visual perception, visual attention and concentration. Previous authors have found that patients with quadriplegia and OSA had deficits in attention, concentration, memory and learning skills [15]. The significance of poor function in these areas is greater in the quadriplegic population, owing to their limited physical functioning. As a result, cognition becomes even more important for optimal participation in rehabilitation and society. Moreover, it is to be expected that performance on these tests will be improved by CPAP treatment.

#### Sleep symptoms and functional consequences of sleepiness

The Basic Nordic Sleep Questionnaire (BNSQ) [22] has been validated in a spinal cord injured population and will provide additional information about sleep quality.

#### Quality of life and health utility values

The Assessment of Quality of Life (AQoL) [23] is a generic quality of life instrument and has been found to be as good as or better than instruments commonly used in measuring outcomes in stroke, co-ordinated care, influenza, cochlear implants, population monitoring and elderly groups. The AQoL provides both health-related quality of life information in the domains of ‘illness’, ‘independent living’, ‘social relationships’, physical senses’, ‘psychological wellbeing’ and a total AQoL value. In addition to these outcomes, the AQoL may be summarized to generate utility values, thereby enabling the generation of quality-adjusted life years (QALY) and facilitating economic analyses.

#### Other tests

In addition, the Hospital Anxiety and Depression Scale (HADS) and the Profile of Mood States (POMS) tests will be used.

### Pre-randomization procedure

All subjects who fulfil the inclusion criteria, are not excluded by the exclusion criteria, consent to participate and are classified with positive OSA will be trialled on auto-titrating CPAP (Resmed AutoSet, San Diego, USA) for up to three nights.

Subjects who are able to use the CPAP for at least four hours on any night will then be randomized as soon as they have succeeded in doing so, into either the treatment or usual care group. If four hours’ use are tolerated on the first night, subjects will be randomized at this time. Those who do not use the CPAP for four hours on any of the three nights will cease participation in the trial. The four-hour cut off was derived from the feasibility trial, where those who were unable to achieve this time were not likely to be compliant over the length of the trial.

The decision to randomize only those who are likely to be adherent with CPAP was the most significant study design modification to arise from the feasibility study. A rate of nonadherence of 50%, as was observed in the feasibility study, would render this study unfeasible. Pre-randomization selection potentially limits the study generalizability to those who are tolerant of CPAP, although it accurately reflects clinical practice (you would never ‘force’ patients to use a therapy they did not accept), was strongly supported by the Australasian Sleep Trials Network, is a similar protocol to that employed in the multinational Sleep Apnoea Cardiovascular Endpoints study [24] and significantly increases the project feasibility by reducing the numbers of subjects, the timeframe and the associated staff cost.

### Randomization

Randomization will be performed centrally using the research study database [25]. A blocked, randomization sequence was generated [26] and loaded into the online database by an independent member of the research unit prior to trial commencement. Allocation concealment is assured through the site design, which requires subject enrolment be completed before randomization can become possible. Additionally, the randomized group is only revealed after a commitment to randomization is both made and confirmed.

### Weekly measures

The following measures will be made at baseline and then weekly upon review:

1. Time and date of assessment.

2. Current medications.

3. Any intercurrent illness.

4. Sleepiness, using the Karolinska Sleepiness Scale (KSS) [27], a ten-item scale, which describes current sleepiness (state).

5. Machine usage data in the CPAP treatment group will be downloaded weekly, and those subjects in the CPAP treatment group will be reviewed with respect to any machine usage or masking issues.

6. Clinical review, autonomic dysfunction assessment and troubleshooting.

All participants will be provided with an autonomic dysfunction symptom diary (Table 1). The diary will record the number, nature and treatment of symptoms typically related to autonomic dysreflexia.

**Table 1:** Autonomic dysreflexia diary

	**Number of events**	**Treated(yes/no)**
Pounding headache, which gets worse		
Flushing and blotching of the skin above the level of the spinal cord injury		
Profuse sweating		
Chills without fever		
Hypertension (high blood pressure)		

In the last week, have you experienced any of the following symptoms, and if so, were they treated?

### Monthly measures

Respiratory function will be measured at baseline and then monthly upon review. All testing will be performed in the supine position. Simple spirometry will be used to obtain vital capacity and forced expiratory volume in 1 second. All tests of respiratory function will be performed without an abdominal binder and in accordance with the performance limitations imposed by quadriplegia [28].

### End study (three months or hospital discharge) data collection

The baseline testing battery will be repeated in the same manner with the exception of the Multivariate Apnoea Prediction Index. Following the completion of all testing procedures, a semistructured subject interview will be held with a subset of the trial participants to examine their experience of the trial and to explore themes related to adherence. The sleep study will be repeated, with the subjects using their CPAP (or not) as randomized. At this time, participants will be provided with full details of their physiological tests and referred for ongoing clinical management of any OSA if they wish.

An adverse event audit will be performed at the conclusion of the study. This process will be overseen by an independent outcomes committee comprising two sleep or spinal specialist clinicians. The patient medical record will be audited for all adverse events defined by the outcomes committee. The blinded assessor at each site will perform the audit. The outcomes committee will review the standardized audit report and will classify any defined event by severity and whether it was or was not likely to have been associated with study treatment or assessment procedures. These classified event reports will be passed to the independent data monitoring and review committee for review.

### Treatment device

Participants in the treatment group will be fitted with a nasal or face mask and head gear. The choice of patient interface will be made locally by the treating teams. The CPAP will be delivered by an auto-titrating device (Resmed AutoSet, San Diego USA). These devices automatically set the level of delivered pressure to ensure upper airway patency, thereby eliminating the need for a pressure titration sleep study to be performed in the sleep laboratory. In addition, if, as was observed in the pilot study, effective CPAP alters over the trial period [19], the device will alter the delivered pressure accordingly to maintain airway patency.

The AutoSet devices record the number and type of respiratory events detected, the pressure delivered in response to these events and the amount of time that the machine was on and delivering pressure to a patient. If the machine is running, but the mask has been removed from the face, this time does not contribute to the measure of CPAP compliance.

### Feasibility, safety and efficacy

The research team have completed a one-year study examining the feasibility and safety of the planned protocol. No major adverse events were observed; in particular, no episodes of excess uncontrolled flow, such that exhalation was impeded by the auto-titrating CPAP were observed [19]. One device was found to be faulty and exchanged. This fault did not affect any participant. All minor adverse events (mask irritation, nasal stuffiness) related to acclimatization to CPAP and are common to all new CPAP users.

### Implementation

A member of the research team will instruct the patients, their families and the ward staff in the use of the CPAP. The ward staff will introduce, apply and adjust the mask for comfort, until the participants are satisfied. The research staff will also review the subjects each morning to identify any barriers to use and address any difficulties. The feasibility study suggested that CPAP adherence could be maximized through early and careful attention to any difficulties.

### Treatment duration

In the able-bodied with OSA, a minimum of 6 weeks of treatment is required to establish CPAP adherence and to observe changes in neuropsychological functioning outcomes. Three months of therapy has been demonstrated as sufficient to improve neurocognitive functioning, specifically improved memory, in the able-bodied with OSA [29]. Other cardiovascular changes occur more slowly; however, improvements would be evident after three months of therapy. We propose to treat people for three months or until hospital discharge.

### Data analysis

All analyses will be performed on an intention-to-treat basis. This presents particular challenges in a clinical trial, such as this, where effective clinician and patient blinding of treatment allocation is impossible.

### Blinding of assessments

In a trial of a physical device such as this, patient and clinical blinding is impossible. We therefore propose to perform blinded assessment of the maximum proportion of the outcome measures. The following measures will be blinded to group allocation:

1. All initial, baseline data, including PSG staging and scoring.

2. Cognitive test battery.

3. Spirometry.

4. Quality of life and sleep questionnaires.

The clinical scenarios outlined below will be managed as described. Both of the first two scenarios occurred during the feasibility trial.

#### Unable to continue with CPAP

Any participant who chooses to cease CPAP treatment will remain in the study for 3 months. The test collection battery will proceed as planned.

#### Hospital discharge prior to end of treatment period

To continue the proposed randomized controlled trial in the home is beyond the scope of the study, therefore the treatment period will cease at hospital discharge. The end study data collection battery will be performed in the week prior to discharge and all obtained results will be treated as ‘last observation carried forward’.

#### Randomized group cross-over

Patients with OSA who are randomized to usual care but then proceed to be treated with CPAP or noninvasive ventilatory support will remain in their allocated group for primary analysis purposes.

### Trial data management

Each site will be responsible for local storage of hard-copy data, which will be entered online into a centralized database. Automated, digital data capture methods will be used wherever possible. Sleep studies will be performed locally and data transferred electronically to the coordinating centre for analysis. This process has been successfully used by the research team in a previous interlaboratory concordance trial [30].

### Estimated subject numbers

This is a three-month treatment trial at a time where there is profound disruption in the life of the study participants. Over this acute period, direct measurements of quality of life and performance in rehabilitation will not demonstrate the efficacy or otherwise of CPAP. The feasibility study clearly demonstrated that there was substantial variability in quality of life post injury, unrelated to OSA or CPAP, thus rendering these outcomes unsuitable for demonstrating short-term improvement in a study sample of constrained size. We are, therefore, basing our sample size calculations on neurocognitive performance, a number of measures of which form our test battery. The specific neurocognitive test in the test battery, which is best characterized in both the spinal population and OSA, is the PASAT. Lower (worse) PASAT scores correlate with sleep fragmentation severity in the able-bodied [31] and OSA severity in quadriplegia [15]. Lower PASAT scores are also associated with diminished frontal lobe function, as assessed by functional magnetic resonance imaging [32] and a mean difference in PASAT scores of seven discriminates between those with and without cognitive impairment in multiple sclerosis [33]. In the able-bodied with OSA, those adherent to CPAP over three months have a PASAT score 18 units higher than those who are not (standard deviation of 33) [34].

Assuming a mean difference between the study arms of 18 [34], a standard deviation of 33, a power of 0.8, an *α* value of 0.05 and a nonadherence to CPAP rate of 15%, 150 subjects will be randomized. To ensure that 150 participants complete the study, we estimate that approximately 820 admissions with quadriplegia will be required across our collaborating centres (at least 8 of 44 admissions will meet the inclusion criteria, fail to meet the exclusion criteria, agree to participate, have OSA and tolerate CPAP). This sample is also adequate to detect any true difference between secondary outcomes, such as sleepiness (KSS number required = 44).

### Adherence with therapy

All usage data collected by the treatment devices will be downloaded weekly and analyzed fully. Based on previous research in the able-bodied population with OSA, improvements in memory, sleepiness and daily function may be observed if CPAP is used for 4 hours per night for five days out of every seven [35,36]. Those subjects who use CPAP for at least this amount will be classified as ‘adherent’ for each week of the study

### Statistical analyses

Usage data from the CPAP devices will be described and subjects classified as adherent, as described. The proportion of subjects continuing to use CPAP over the three months of treatment will be plotted and examined using survival (Kaplan-Meier) analysis. Statistics describing the distribution of CPAP usage, average numbers of hours used per night, and so on, will be calculated.

Changes in neurocognitive performance, AQoL, BNSQ and KSS will be examined with paired *t* tests, repeated measures analysis of variance and generalized linear mixed models, as appropriate. The rate of autonomic dysreflexia and the time course of development will be compared with *χ* squared and mixed model regression modelling as appropriate. Exploratory linear and logistic regression modelling will be performed to determine whether the average number of hours used or the likelihood of adherence can be predicted by any of the groups’ baseline characteristics.

### Project governance and administrative support

The chief investigator (Dr Berlowitz) will be responsible for overall project management, but is assisted and advised by a project steering committee comprised of the collaborating researchers and administrative support from the administering coordinating institution. Additional support and membership has been seconded from the partner organizations. The project steering committee will meet regularly and all agendas and minutes circulated to all stakeholders. Funding agreements will be entered into between all collaborating agencies and the administering institution. The project will be supported by a part-time project manager.

An outcomes committee will be established as detailed above and an independent Data Monitoring Committee will be externally appointed by the Australasian Clinical Trials Network.

### Follow-up at trial completion

Participants in the treatment group will be given their mask and CPAP device at the end of the 3 months to enable them to continue treatment of their OSA. Those randomized to the control group will be fully supported to commence CPAP treatment and also offered a mask and CPAP device for ongoing use. All participants will be referred to their local respiratory or sleep clinic as appropriate for ongoing management of their OSA.

## Discussion

This trial will determine the effectiveness of nocturnal CPAP in the acute quadriplegic population. In particular, improvement in cognitive performance in this group may improve their ability to engage with the rehabilitation process and allow them to participate more fully in life following the initial rehabilitation phase. The profound reduction in physical functioning that characterizes quadriplegia means a greater reliance on cognitive functioning for work and participation in family and community life post injury. Any therapy that optimizes cognitive function is potentially of great significance to those with quadriplegia, their families, friends and colleagues.

## Trial status

The trial commenced recruitment in July 2009. Recruitment will cease when 150 trial participants have been randomized. It is anticipated that this target will be reached by mid-2015.

## Abbreviations

AQoL: Assessment of Quality of Life; BNSQ: Basic Nordic Sleep Questionnaire; CPAP: Continuous positive airway pressure; ECG: Electrocardiography; HADS: Hospital Anxiety and Depression Scale; KSS: Karolinska Sleepiness Scale; OSA: Obstructive sleep apnoea; PASAT: Paced Auditory Serial Addition Test; POMS: Profile of Mood States; SCI: Spinal cord injury; QALY: Quality-adjusted life years.

## Competing interests

David Berlowitz has received competitive research funding support from the ResMed Foundation in the USA. Additionally, he has received competitive research support from the Transport Accident Commission to perform research examining other aspects of sleep and respiratory disorders in spinal cord injury. He declares that he has no other financial or nonfinancial competing interests. Douglas Brown has received competitive research support from the Transport Accident Commission to perform research in spinal cord injury. He declares that he has no other financial or nonfinancial competing interests. Peter Cistulli has received research support from ResMed Inc. (CPAP) and SomnoMed Ltd (oral appliances) for investigator-initiated studies in obstructive sleep apnoea. He has served on SomnoMed's medical advisory board and has an ongoing pecuniary interest in the company. He is currently a medical advisor to ExploraMed (a medical device incubator). Fergal O’Donoghue has received competitive research support from the Transport Accident Commission to perform research examining other aspects of sleep and respiratory disorders in spinal cord injury. He declares that he has no other competing financial or nonfinancial competing interests. Najib Ayas, Maree Barnes, Tim Geraghty, Alison Graham, Bonne Lee, Meg Morris, Peter Rochford, Jack Ross, Raj Singhal, Jo Spong and Brooke Wadsworth declare that they have no competing interests. Robert Pierce has passed away since protocol development.

## Authors’ contributions

The trial protocol was developed by all authors during a two-day workshop in Melbourne, Australia in June 2008 from an original idea developed by DB, DB and RP. DB and JR were responsible for initial manuscript preparation. All authors reviewed the final version prior to submission.

## References

[B1] O’Connor PJ Cripps RA Spinal Cord Injury Australia, 1997/98 1998 Research Centre for Injury Studies: Flinders University of South Australia

[B2] O’Connor PJ Spinal Cord Injury, Australia 1998/99 2000 Research Centre for Injury Studies: Flinders University of South Australia

[B3] Access Economics The Economic Cost of Spinal Cord Injury and Traumatic Brain Injury in Australia 2009 Canberra, Australia: Report by Access Economics Pty Limited for the Victorian Neurotrauma Initiative

[B4] Short DJ Stradling JR Williams SJ Prevalence of sleep apnoea in patients over 40 years of age with spinal cord lesions J Neurol Neurosurg Psychiatry 1992 55 1032 1036 10.1136/jnnp.55.11.1032 1469399 PMC1015288

[B5] McEvoy RD Mykytyn I Sajkov D Flavell H Marshall R Antic R Thornton AT Sleep apnoea in patients with quadriplegia Thorax 1995 50 613 619 10.1136/thx.50.6.613 7638801 PMC1021258

[B6] Biering-Sorensen F Biering-Sorensen M Hilden J Reproducibility of Nordic Sleep Questionnaire in spinal cord injured Paraplegia 1994 32 780 786 10.1038/sc.1994.124 7885721

[B7] Burns SP Little JW Hussey JD Lyman P Lakshminarayanan S Sleep apnea syndrome in chronic spinal cord injury: associated factors and treatment Arch Phys Med Rehabil 2000 81 1334 1339 10.1053/apmr.2000.9398 11030498

[B8] Levi R Hultling C Nash MS Seiger A The Stockholm spinal cord injury study: 1. Medical problems in a regional SCI population Paraplegia 1995 33 308 315 10.1038/sc.1995.70 7644255

[B9] Star AM Osterman AL Sleep apnea syndrome after spinal cord injury. Report of a case and literature review Spine 1988 13 116 117 10.1097/00007632-198801000-00029 3381121

[B10] Stockhammer E Tobon A Michel F Eser P Scheuler W Bauer W Baumberger M Muller W Kakebeeke TH Knecht H Zach GA Characteristics of sleep apnea syndrome in tetraplegic patients Spinal Cord 2002 40 286 294 10.1038/sj.sc.3101301 12037710

[B11] Berlowitz DJ Spong J Gordon I Howard ME Brown DJ Relationships between objective sleep indices and symptoms in a community sample of people with tetraplegia Arch Phys Med Rehabil 2012 93 1246 1252 10.1016/j.apmr.2012.02.016 22516876

[B12] Young T Palta M Dempsey J Skatrud J Weber S Badr S The occurrence of sleep-disordered breathing among middle-aged adults N Engl J Med 1993 328 1230 1235 10.1056/NEJM199304293281704 8464434

[B13] Berlowitz DJ Brown DJ Campbell DA Pierce RJ A longitudinal evaluation of sleep and breathing in the first year after cervical spinal cord injury Arch Phys Med Rehabil 2005 86 1193 1199 10.1016/j.apmr.2004.11.033 15954059

[B14] Tran K Hukins C Geraghty T Eckert B Fraser L Sleep-disordered breathing in spinal cord-injured patients: a short-term longitudinal study Respirology 2010 15 272 276 10.1111/j.1440-1843.2009.01669.x 19947995

[B15] Sajkov D Marshall R Walker P Mykytyn I McEvoy RD Wale J Flavell H Thornton AT Antic R Sleep apnoea related hypoxia is associated with cognitive disturbances in patients with tetraplegia Spinal Cord 1998 36 231 239 10.1038/sj.sc.3100563 9589522

[B16] Kingshott RN Vennelle M Hoy CJ Engleman HM Deary IJ Douglas NJ Predictors of improvements in daytime function outcomes with CPAP therapy Am J Respir Crit Care Med 2000 161 866 871 10.1164/ajrccm.161.3.9905053 10712335

[B17] Barnes M Houston D Worsnop CJ Neill AM Mykytyn IJ Kay A Trinder J Saunders NA Douglas McEvoy R Pierce RJ A randomized controlled trial of continuous positive airway pressure in mild obstructive sleep apnea Am J Respir Crit Care Med 2002 165 773 780 10.1164/ajrccm.165.6.2003166 11897643

[B18] Burns SP Rad MY Bryant S Kapur V Long-term treatment of sleep apnea in persons with spinal cord injury Am J Phys Med Rehabil 2005 84 620 626 10.1097/01.phm.0000171008.69453.b9 16034232

[B19] Berlowitz DJ Spong J Pierce RJ Ross J Barnes M Brown DJ The feasibility of using auto-titrating continuous positive airway pressure to treat obstructive sleep apnoea after acute tetraplegia Spinal Cord 2009 47 868 873 10.1038/sc.2009.56 19488050

[B20] Maislin G Pack AI Kribbs NB Smith PL Schwartz AR Kline LR Schwab RJ Dinges DF A survey screen for prediction of apnea Sleep 1995 18 158 166 7610311 10.1093/sleep/18.3.158

[B21] Flemons WW Buysse D Redline S Pack A Sleep-related breathing disorders in adults: recommendations for syndrome definition and measurement techniques in clinical research. The Report of an American Academy of Sleep Medicine Task Force Sleep 1999 22 667 689 10450601

[B22] Biering-Sorensen F Biering-Sorensen M Sleep disturbances in the spinal cord injured: an epidemiological questionnaire investigation, including a normal population Spinal Cord 2001 39 505 513 10.1038/sj.sc.3101197 11641793

[B23] Hawthorne G Richardson J Osborne R The Assessment of Quality of Life (AQoL) instrument: a psychometric measure of health-related quality of life Qual Life Res 1999 8 209 224 10.1023/A:1008815005736 10472152

[B24] Sleep Apnoea Cardiovascular Endpoints Study (SAVE) http://www.savetrial.org

[B25] Sleep Health in Quadriplegia http://www.shiq.com.au

[B26] Randomization.com http://www.randomization.com

[B27] Gillberg M Kecklund G Akerstedt T Relations between performance and subjective ratings of sleepiness during a night awake Sleep 1994 17 236 241 7939123 10.1093/sleep/17.3.236

[B28] Ashba J Garshick E Tun CG Lieberman SL Polakoff DF Blanchard JD Brown R Spirometry–acceptability and reproducibility in spinal cord injured subjects J Am Paraplegia Soc 1993 16 197 203 8270915 10.1080/01952307.1993.11735901

[B29] Zimmerman ME Arnedt JT Stanchina M Millman RP Aloia MS Normalization of memory performance and positive airway pressure adherence in memory-impaired patients with obstructive sleep apnea Chest 2006 130 1772 1778 10.1378/chest.130.6.1772 17166995

[B30] Rochford P Ruehland W Pierce R Singh P Thornton A Inter-observer variability in PSG scoring in a large Australasian dataset Sleep Biol Rhythms 2007 5 A44

[B31] Martin S Engleman H Deary I Douglas N The effect of sleep fragmentation on daytime function Am J Respir Crit Care Med 1996 153 1328 1332 10.1164/ajrccm.153.4.8616562 8616562

[B32] Cardinal KS Wilson S Giesser BS Drain AE Sicotte NL A longitudinal fMRI study of the paced auditory serial addition task Mult Scler 2008 14 465 71 10.1177/1352458507084263 18208900

[B33] Rosti E Hamalainen P Koivisto K Hokkanen L The PASAT performance among patients with multiple sclerosis: analyses of responding patterns using different scoring methods Mult Scler 2006 12 586 593 10.1177/1352458506070624 17086904

[B34] Felver-Gant JC Bruce AS Zimmerman M Sweet LH Millman RP Aloia MS Working memory in obstructive sleep apnea: construct validity and treatment effects J Clin Sleep Med 2007 3 589 594 17993040 PMC2045718

[B35] Weaver TE Maislin G Dinges DF Bloxham T George CFP Greenberg H Kader G Mahowald M Younger J Pack AI Relationship between hours of CPAP use and achieving normal levels of sleepiness and daily functioning Sleep 2007 30 711 719 17580592 10.1093/sleep/30.6.711 PMC1978355

[B36] Weaver TE Grunstein RR Adherence to continuous positive airway pressure therapy: the challenge to effective treatment Proc Am Thorac Soc 2008 5 173 178 10.1513/pats.200708-119MG 18250209 PMC2645251

